# Prediction of pathological and oncological outcomes based on extended prostate biopsy results in patients with prostate cancer receiving radical prostatectomy: a single institution study

**DOI:** 10.1186/1746-1596-7-68

**Published:** 2012-06-14

**Authors:** Fumio Ishizaki, Noboru Hara, Hiroshi Koike, Makoto Kawaguchi, Akira Tadokoro, Itsuhiro Takizawa, Tsutomu Nishiyama, Kota Takahashi, Rudolf Hohenfellner

**Affiliations:** 1 Department of Urology, Joetsu, 942-8502, Japan; 2 Pathology, Niigata Rousai Hospital, Joetsu, 942-8502, Japan; 3 Division of Urology, Department of Regenerative and Transplant Medicine, Graduate School of Medical and Dental Sciences, Niigata University, Nigata, Japan; 4 Department of Urology, Johannes Gutenberg University, Mainz, Germany

**Keywords:** Extended prostate biopsy, Seminal vesicle involvement, Positive surgical margin, Perineural invasion

## Abstract

**Background:**

The prediction of pathological outcomes prior to surgery remains a challenging problem for the appropriate surgical indication of prostate cancer. This study was performed to identify preoperative values predictive of pathological and oncological outcomes based on standardized extended prostate biopsies with core histological results diagrammed/mapped in patients receiving radical prostatectomy for prostate cancer clinically diagnosed as localized or locally advanced disease.

**Methods:**

In 124 patients with clinically localized or locally advanced prostate cancer (cT1c–cT3a) without prior treatment, pathological outcomes on the surgical specimen including seminal vesicle involvement (SVI), positive surgical margin (PSM), and perineural invasion (PNI) were studied in comparison with clinical parameters based on the results of 14-core prostate biopsies comprising sextant, laterally-directed sextant, and bilateral transition zone (TZ) sampling.

**Results:**

Concerning the association of pathological outcomes with oncological outcomes, patients with PSM and PNI on surgical specimens had poorer biochemical-progression-free survival than those without PSM (logrank p = 0.002) and PNI (p = 0.003); it was also poorer concerning SVI, although the difference was not significant (p = 0.120). Concerning the impact of clinical parameters on these pathological outcomes, positive TZ and multiple positive biopsy cores in the prostatic middle were independent values predictive of SVI with multivariate analyses (p = 0.020 and p = 0.025, respectively); both positive TZ and multiple positive prostatic middle biopsies were associated with larger tumor volume (p < 0.001 in both). The percentage of positive biopsy cores (%positive cores) and biopsy Gleason score were independent values predictive of PSM (p = 0.001) and PNI (p = 0.001), respectively. Multiple positive cores in the prostatic base were associated with proximal/bladder-side PSM (p < 0.001), and also linked to poorer biochemical-progression-free survival (p = 0.004). Clinical T stage had no association with these pathological outcomes.

**Conclusions:**

%positive cores and Gleason score in extended biopsies were independent values predictive of PSM and PNI in prostate cancer clinically diagnosed as localized or locally advanced disease, respectively, which were associated with poorer oncological outcomes. When diagramming biopsy-core results, extended biopsy may provide additional information for predicting oncological and pathological outcomes including SVI in patients clinically diagnosed as having localized or locally advanced disease.

**Virtual slides:**

The virtual slide(s) for this article can be found here: http://www.diagnosticpathology.diagnomx.eu/vs/8790262771042628

## Background

Since the advent of the preoperative staging table/nomogram, the clinical staging of localized or locally advanced prostate cancer has been revolutionized both for radical surgery and radiotherapy [1-3]. These predictive scales are used for the calculation of possible oncological outcomes or probability of extraprostatic extension in the pathological stage, for example, seminal vesicle involvement (SVI), by combining clinical parameters/variables such as serum variable prostate-specific antigen levels, digital rectal examination findings, Gleason score at prostate biopsy, and percentage of positive biopsy cores (%positive cores) [1-3]. Although extraprostatic disease does not necessarily indicate incurable disease, SVI has been associated with poor oncological outcomes [3-6]. However, advances in the aforementioned staging modalities have led to the low prevalence of SVI in pathological outcomes, and it has been reported that the recent low prevalence of SVI makes the validation of prognostic models difficult [7]. On the other hand, positive surgical margin (PSM) and perineural invasion (PNI) have also been suggested to have a prognostic value [6,8], but clinical parameters predictive of PSM remain controversial and studies on PNI are limited. The prediction of these pathological outcomes prior to surgery is significant in clinical practice, but there has been no study examining which clinical parameters reflect each of SVI, PSM, and PNI; such analyses may confirm the feasibility of staging nomograms also based on pathological approaches.

Several previous studies assessed pathological outcomes such as SVI in comparison with observations using conventional sextant to octant biopsies [9,10], but there has been no study examining predictive values for the mentioned pathological outcomes based on extended/extensive prostate biopsies that may potentially provide more accurate information concerning the disease condition and extension [11]. The aim of the present study was to identify preoperative clinicopathological factors associated with SVI, PSM, and PNI, based on standardized extended prostate biopsies with core histological results precisely diagrammed in men who underwent radical prostatectomy for prostate cancer clinically diagnosed as localized or locally advanced disease. In addition, oncological outcomes were assessed to clarify whether such factors identified based on extended biopsy results also have a prognostic value in them.

## Methods

### Patients

We reviewed 144 consecutive patients, who were diagnosed with prostate cancer based on standardized 14-core prostate biopsy described elsewhere and treated with standardized radical prostatectomy with standard or modified pelvic lymphadenectomy for localized or locally advanced prostate cancer (cT1c–3a N0 M0) at our institution between June 2003 and March 2010. Twenty patients who received prior therapy such as androgen deprivation or radiotherapy were excluded. The final study group comprised 124 patients. All surgical procedures were performed by an experienced urologist (HK) with assistance by experienced urologists (NH and AT). The procedure for this research project was approved by the Ethics Committee of our institution. Informed consent was obtained from all patients.

Clinical stages were determined according to the International Union Against Cancer (UICC) classification of 2009. Clinical staging routinely included abdominal and pelvic computerized tomography (CT), chest radiograph or thoracic CT, isotope bone scanning, and extended/extensive prostate biopsy, as described elsewhere. Patients’ demographics are shown in Table 1. PSA levels at diagnosis ranged between 2.6 and 63.7 (mean: 9.0 ng/ml), and Gleason score at biopsy ranged between 6 and 10. Biochemical progression/PSA failure after surgery was defined as 2 consecutive PSA values of 0.2 ng/ml or greater at any time postoperatively, or any additional treatment more than 6 months after radical prostatectomy [12]. Five patients receiving adjuvant androgen deprivation therapy immediately after surgery were excluded from survival analyses. The mean and median observation periods were 31.0 and 24.0 months, respectively.

**Table 1:** Patients’ characteristics (n = 124)

**Age at diagnosis, mean (range)**	**63.8 (40 – 73)**
PSA levels at diagnosis (ng/ml)	No. of patients
< 4.0	13 (10.5%)
4.0 – 10.0	75 (60.5%)
10.1 – 20.0	29 (23.4%)
20.1 – 50.0	6 (4.8%)
50.1 or higher	1 (0.8%)
Gleason score at biopsy	No. of patients
6	48 (38.7%)
3 + 4	20 (16.1%)
4 + 3	20 (16.1%)
8 or higher	36 (29.0%)

PSA, prostate-specific antigen.

### Prostate biopsy

**Figure 1:**
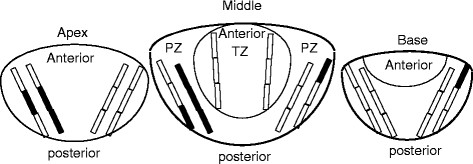
**Histological localization diagram in a 67 year-old man with 6 positive cores in 14-core prostate biopsy, showing positive segments in cores as bars painted in black (TZ, transition zone; PZ, peripheral zone); each core was histologically assessed in 3 divided core segments**.

Transrectal 14-core systemic prostate biopsies were carried out using transrectal ultrasound Aloka SSD 3300 CV (Aloka, Tokyo, Japan) following the method of standard sextant biopsy, in addition to those for sextant laterally directed biopsy and bilateral single core transition zone biopsy, as described previously [13]; 14 cores thus comprised 4 cores for each of the prostatic base, middle, and apex, and 2 cores for the transition zone. We separated the patients to subgroups based on those with a single positive core or multiple positive cores in each of the prostatic apex, middle, and base; this dichotomization was determined based on previous studies reporting that a single positive core in sextant or less biopsies is a requirement for categorizing to low-risk disease [14]. The maximal core length obtained by needle biopsy was 22 mm; the mean measured core length was 15.9 ± 3.2 mm. Each core was tagged and mapped to produce histological localization diagrams (Figure 1).

### Histopathological diagnosis

An independent pathologist (MK) made all histopathological diagnoses, and standardized grading was carried out according to the contemporary Gleason classification system [15]. All pathological examinations were performed using the whole-mount step-section technique. The prostate was weighed, measured, and inked, and was subsequently fixed in buffered formalin for at least 24 hours before sectioning. Following fixation, the specimen was step-sectioned, and was submitted in entire cross-sections from the apex to base at 3 – 5-mm intervals; the mean number of sections was 27.1 ± 6.6. The apex and bladder neck were coned and serially sectioned. Cancer foci were also diagrammed in photographs of the sectioned specimen. SVI was defined following the criteria of Epstein et al., as the invasion to the muscular wall of the seminal vesicle [16]. PNI in the radical prostatectomy specimens was assessed following the method reported previously [17-19]. Surgical margins were assessed adopting an approach similar to that in the recent consensus criteria [20].

### Statistical analysis

In addition to the chi-square and Fisher's exact tests, the Mann–Whitney *U* test was used to compare unpaired parameters between two subgroups. Independent predictive values were identified with multivariate analysis using a logistic regression model; variables having p-value of p < 0.1 in univariate analyses were tested with multivariate analysis. Statistical analysis of Gleason score was performed with a stepwise model. Survival curves were generated using the method of Kaplan and Meier, and differences between curves were evaluated employing the log-rank test. Uni- and multivariate analyses for survival-associated parameters were performed using Cox proportional hazard models. Correlations between parameters were analyzed using Spearman’s rank correlation coefficient (rs) analysis. The test was two-sided except for the Fisher's exact test, and p < 0.05 was considered significant. All analyses were performed using SPSS version 15.0 J (SPSS Inc., Chicago, IL, USA) on a Windows-based computer.

## Results

### Pathological stage and Gleason score in radical prostatectomy specimens

There were 81 (65.3%) with pT2 and 42 (33.9%) patients with pT3 disease. Thirty-six (29.3%) and 4 (3.2%) of the patients experienced pathological upstaging from and downstaging, respectively. One patient had pT0 disease. Also, there were 48 (38.7%) with pathological Gleason score of 6, 40 (32.3%) with pathological Gleason score of 7, and 36 (29.0%) patients with pathological Gleason score of 8 or higher. Forty-eight (39.0%) and 27 (22.0%) of the patients experienced upgrade and downgrade of Gleason score, respectively.

### Relationship between SVI and clinical parameters or biopsy findings

Table 2 shows the relationships between the presence/absence of SVI and clinical parameters or tumor localizations based on prostate biopsies. PSA levels, %positive cores, and the cT stage were not different between those with and without SVI. Patients with SVI showed a higher Gleason score at biopsy, higher rate of multiple (2 or more) positive cores in the prostatic middle, and a higher transition zone-positive rate, compared with those without SVI (p = 0.005, p = 0.005, and p = 0.002, respectively); with multivariate analyses using the logistic regression model, positive transition zone (adjusted odds ratio: 12.99 [95%CI: 1.490–111.1], p = 0.023) and multiple positive biopsies in the prostatic middle (adjusted odds ratio: 7.299 [95%CI: 1.312–40.00], p = 0.025) were independent values predictive of seminal vesicle involvement.

**Table 2:** Variance in preoperative clinicopathological parameters between patients with and without seminal vesicle involvement (SVI)

	**SVI present** **(n = 8)**	**SVI absent** **(n = 116)**	**Univariate**	**Multivariate**	
			**P value**	**P value**	**Adjusted odds ratio (95% CI)**
Mean age [years]	63.6 ± 3.9	63.9 ± 5.1	0.725		
PSA [ng/ml]	13.0 ± 8.1	8.7 ± 7.2	0.105		
Gleason score at biopsy	8.1 ± 1.1	7.0 ± 1.0	0.005		
cT stage (n)			0.153		
T1c	5 (62.5%)	94 (81.0%)			
T2	3 (37.5%)	15 (12.9%)			
T3a	0 (0%)	7 (6.0%)			
%positive cores	31.0 ± 21.3	21.3 ±16.4	0.242		
TZ biopsy positive (n)	7 (87.5%)	35 (30.2%)	0.002	0.023	12.99 (1.490-111.1)
Number of apex-positive (2–4/0–1) (n)	3/5	26/90	0.278		
Number of middle-positive (2–4/0–1) (n)	6/2	28/88	0.005	0.025	7.299 (1.312-40.00)
Number of base-positive (2–4/0–1) (n)	4/4	23/93	0.067		

PSA, prostate-specific antigen; TZ, transition zone; apex, prostatic apex biopsy; middle, prostatic middle biopsy; base, prostatic base biopsy.

Results when analyzing data on each of the standard sextant sites or laterally-directed sextant cores had no association with pathological outcomes (data not shown). When assessed with tumor localization in radical prostatectomy specimens, the positive-predictive value of TZ biopsy was 0.86; 86% of the patients with positive TZ biopsy also had TZ cancer or cancer invading to TZ in surgical specimens. All of the patients with positive prostatic middle biopsy also had cancer foci in the prostatic middle in surgical specimens. The largest tumor size ranged between 2 – 45 (mean: 16.8 ± 9.4) mm (pT0 in one patient) in prostatectomy specimens; patients with a positive TZ biopsy had a larger tumor size than those with a negative TZ biopsy (mean 21.5 ± 10.2 mm *vs.* 14.2 ± 7.8 mm, respectively, P < 0.001). Also, patients with multiple positive cores in prostatic middle biopsy had a larger tumor size than those with no or a single positive core in prostatic middle biopsy (mean: 22.0 ± 11.3 mm *vs.* 14.9 ± 7.7 mm, respectively, P < 0.001).

### Relationship between PSM and clinical parameters or biopsy findings

Table 3 shows the relationships between the presence/absence of PSM and clinical parameters or tumor localizations based on prostate biopsies. Data on the surgical margin were not available in 4 patients. PSA levels, biopsy Gleason score, and cT stage were not different between those with and without PSM. Patients with PSM showed higher %positive cores and a higher rate of multiple positive cores in the prostatic base, middle, and apex, compared with those without PSM (p = 0.001, 0.049, 0.004, and 0.037, respectively); with multivariate analyses using the logistic regression model, %positive cores was an independent value predictive of PSM (adjusted odds ratio: 1.044 [95%CI: 1.019-1.070], p = 0.001, Table 4). %positive cores was also correlated with the largest tumor size in prostatectomy specimens (rs = 0.354, p < 0.001).

**Table 3:** Variance in preoperative clinicopathological parameters between patients with and without positive surgical margin (PSM)

	**PSM present** **(n = 39)**	**PSM absent** **(n = 81)**	**Uniivariate**	**Multivariate**	
			**p-value**	**P value**	**Adjusted odds ratio (95% CI)**
Mean age [years]	63.5 ± 36.0	64.0 ± 4.6	0.993		
PSA [ng/ml]	9.8 ± 6.3	8.7 ± 7.8	0.247		
Gleason score at biopsy	7.2 ± 1.2	7.0 ± 1.0	0.209		
cT stage (n)			0.347		
T1c	30 (76.9%)	65 (80.2%)			
T2	5 (12.8%)	13 (16.0%)			
T3a	4 (10.3%)	3 (3.7%)			
%positive cores	31.0 ± 21.3	21.3 ±16.4	0.001	0.001	1.044 (1.019-1.070)
TZ biopsy positive (n)	17 (43.6%)	24 (29.6%)	0.131		
Number of apex-positive (2–4/0–1) (n)	14/25	15/66	0.037		
Number of middle-positive (2–4/0–1) (n)	17/22	15/66	0.004		
Number of base-positive (2–4/0–1) (n)	13/26	14/67	0.049		

PSA, prostate-specific antigen; TZ, transition zone; apex, prostatic apex biopsy; middle, prostatic middle biopsy; base, prostatic base biopsy. Data not available in 4 patients.

**Table 4:** Variance in preoperative clinicopathological parameters between patients with and without peruneural invasion (PNI)

	**PNI present** **(n = 63)**	**PNI absent** **(n = 61)**	**Univariate**	**Multivariate**	
			**P value**	**P value**	**Adjusted odds ratio (95% CI)**
Mean age [years]	63.7 ± 5.0	64.0 ± 5.0	0.894		
PSA [ng/ml]	9.5 ± 5.9	8.5 ± 8.5	0.104		
Gleason score at biopsy	7.4 ± 1.1	6.7 ± 0.9	<0.001	0.001	1.983 (1.342-2.929)
cT stage (n)			0.615		
T1c	49 (77.8%)	50 (82.0%)			
T2	9 (14.3%)	9 (14.8%)			
T3a	5 (7.9%)	2 (3.3%)			
%positive cores	23.5 ± 18.7	19.8 ±14.8	0.513		
TZ biopsy positive (n)	24 (38.1%)	18 (29.5%)	0.312		
Number of apex-positive (2–4/0–1) (n)	14/49	15/46	0.755		
Number of middle-positive (2–4/0–1) (n)	21/42	13/48	0.134		
Number of base-positive (2–4/0–1) (n)	17/46	10/51	0.153		

PSA, prostate-specific antigen; TZ, transition zone; apex, prostatic apex biopsy; middle, prostatic middle biopsy; base, prostatic base biopsy.

### Relationship between PNI and clinical parameters or biopsy findings

Table 4 shows the relationships between the presence/absence of PNI and clinical parameters or tumor localizations based on prostate biopsies. Gleason score on biopsy alone was higher in patients with than in those without PNI (univariate p < 0.001, multivariate p = 0.001, adjusted odds ratio: 1.983 [95%CI: 1.342-2.929]).

### Oncological outcomes

**Figure 2:**
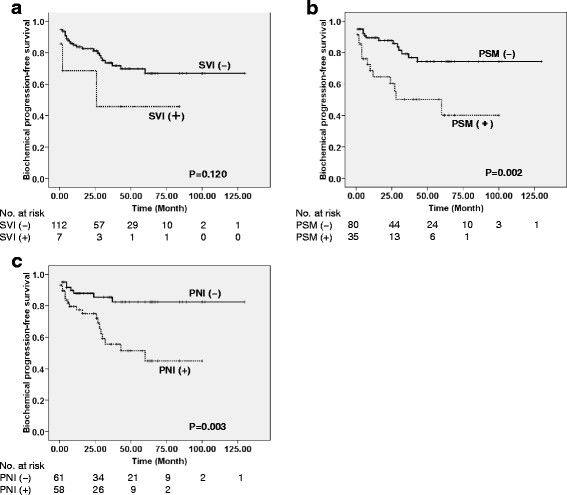
Biochemical-progression-free survival in patients with or without seminal vesicle involvement (SVI, A), a positive surgical margin (PSM, B), and perineural invasion (PNI, C).

**Figure 3:**
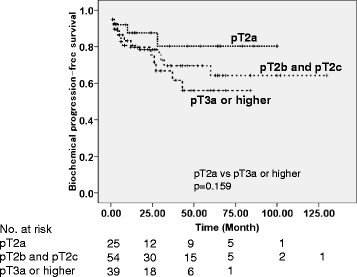
Biochemical-progression-free survivals categorized according to pathological T (pT) stage.

Five men receiving immediate adjuvant androgen deprivation therapy and those with missing data were excluded from survival analyses to assess appropriate biochemical progression. Regarding pathological outcomes, patients with PSM had poorer biochemical-progression-free survival than those without PSM (logrank p = 0.002), and this was also the case for PNI (p = 0.003) (Figure 2). Although patients with SVI seemingly showed a lower biochemical-progression-free survival rate than those without SVI, the difference between survival curves were not significant (logrank p = 0.120) (Figure 2). Among preoperative clinical parameters, patients with PSA levels of 10 ng/ml or higher had poorer biochemical-progression-free survival than those with PSA levels less than 10 ng/ml (p = 0.033). Biochemical-progression-free survival was lower in patients with biopsy Gleason score of 8 or higher than in those with Gleason score of < =7 (p = 0.027). Patients with %positive cores of > = 35.7% (number of positive cores of 5 or more) showed poorer biochemical-progression-free survival than those with %positive cores of < 35.7% (p = 0.023). Patients with multiple positive cores in the prostatic base had poorer biochemical-progression-free survival (p = 0.004); patients with multiple positive cores in the prostatic base more frequently had proximal/bladder-side PSM than those with a single or no positive core in the prostatic base (48.1 *vs.* 6.5%, respectively, p < 0.001). We additionally showed biochemical-progression-free survivals according to pT stage (Figure 3).

## Discussion

In the present study, PSM was a significant pathological factor associated with poorer oncological outcomes, and %positive cores was an independent value predictive of PSM (multivariate p = 0.001) showing a prognostic significance (p = 0.023) in patients with prostate cancer clinically diagnosed as localized or locally advanced disease. These results suggest that %positive cores based on extended biopsies may be useful for predicting oncological outcomes. %positive cores theoretically reflects the tumor volume [21], and thus is a feasible index for predicting PSM. In addition, patients with multiple positive cores in the prostatic base had poorer oncological outcomes, and they more frequently had proximal/bladder-side PSM than those with a single or no positive core in the prostatic base (48.1 *vs.* 6.5%, respectively, p < 0.001). Several previous studies have reported that proximal-side PSM is an adverse prognostic factor [22], and it has also been shown that positive cores in the prostatic base are associated with a higher rate of proximal PSM [23], but the relevant previous studies did not perform survival analyses. The present study additionally suggested that multiple positive cores in the prostatic base with extended biopsy protocol possibly have a prognostic significance, although further studies are required to draw a conclusion.

Previous studies have reported the frequency of PNI in radical prostatectomy specimens to be between 32 and 79%. [19-21], and it was about 50% in the present study (Table 3). A few studies reported that the presence of PNI is associated with poor oncological outcomes [8], and our study also supported the prognostic value of PNI. The current study also suggested that biopsy Gleason score was an independent value predictive of PNI. It has been shown that PNI of prostate cancer is relevant to a tumor’s invasive capacity [24], while Gleason score is also involved in invasiveness of prostate cancer in relation to the expression of matrix metalloproteinases [25,26]. A study regarding the diagnostic value of PNI in extended biopsy cores is currently underway.

Recent advances in staging modalities and surgical indications have been associated with the low prevalence of SVI in surgical outcomes, and it has been reported that the recent low prevalence of SVI makes the validation of prognostic models/nomogram difficult [7]. SVI did not have a prognostic significance in the present study most probably due to the small number of those with SVI; the survival curves categorized with and without SVI seemingly separated (Figure 2). Additionally, the 5 patients excluded from survival analyses because of immediate inception of adjuvant androgen deprivation therapy showed SVI, and their exclusion probably contributed to the absence of statistical significance for SVI. There has been no study examining associations between these pathological conditions and the results of precisely profiled extended biopsy. When mapping/diagramming positive cores, extended biopsy may be of value to predict SVI in patients with prostate cancer clinically diagnosed as localized disease. Interestingly, the number/percentage of positive cores calculated for the standard sextant sites or laterally-directed sextant cores did not reflect SVI, whereas positive TZ and multiple positive cores in the prostatic middle were independently associated with SVI. However, positive-predictive value of TZ biopsy assessed with tumor localization in the prostatectomy specimen was 0.86, suggesting contaminations of cancer arising from the peripheral zone. Patients with a positive TZ biopsy or multiple positive cores in prostatic middle biopsy had a larger tumor size in prostatectomy specimens. With the current 14-core biopsy protocol, parameters such as positive TZ and multiple positive cores in the prostatic middle possibly reflect the tumor size, and thus are associated with SVI. To validate the significance of these observations, studies comparing various biopsy protocols are needed.

In the present study with the mentioned extended biopsy protocol, positive TZ and multiple positive cores in the prostatic middle were independently correlated with SVI (p = 0.020 and p = 0.023, respectively). Also, %positive cores was an independent factor predictive of PSM (p = 0.001), and biopsy Gleason score was independently correlated with PNI (p = 0.001). Thus, clinical factors predictive of pathological outcomes varied among SVI, PSM, and PNI; these results suggest that prevailing staging nomograms or risk-assessment scoring systems combining multiple values such as Gleason score and %positive cores are practically possible based on the pathological approach employing extended biopsy protocols [1-3]. Additionally, artificial intelligence techniques such as artificial neural networks incorporating mentioned informations obtained from extended biopsies potentially support decision-making in prostate cancer treatment [27].

In this study, one patient (0.8%) had pT0 disease, and the frequency was similar to those in previous reports [28]. The present study had several limitations. The study was retrospectively designed, and the relatively small number of patients may have led to a limited statistical power. Also, the small number of locally advanced disease cases is a limitation of this type of study; surgical indication probably affected the background of the study group, as discussed elsewhere. Further follow-up studies are warranted to confirm the significance of the observations on oncological outcomes in men with localized prostate cancer.

## Conclusions

PSM and PNI were significant pathological factors associated with poorer oncological outcomes, and %positive cores and Gleason score in extended biopsies were independent values predictive of PSM and PNI in patients with prostate cancer clinically diagnosed as localized disease, respectively. When mapping/diagramming positive cores, extended prostate biopsies may provide additional value in the prediction of oncological and pathological outcomes such as SVI; multiple positive cores in the prostatic base possibly lead to poorer oncological outcomes.

## Abbreviations

CT, Computerized tomography; PSA, Prostate-specific antigen; TZ, Transition zone; SVI, Seminal vesicle involvement; PSM, Positive surgical margin; PNI, Perineural invasion.

## Competing interests

The authors declare that they have no competing interests.

## Authors’ contributions

FI performed data analysis and helped to draft the manuscript. NH wrote the manuscript and supervised throughout the study. HK carried out all surgical procedures and participated in data collection. MK performed all histopathological diagnoses. AT assisted surgery, clinical management of patients, and data collection. IT and TN helped data analysis. KT assisted to draft the manuscript. RH was a supervisor of this study. All authors read and approved the final manuscript.
